# Decreased Count and Dysfunction of Circulating EPCs in Postmenopausal Hypercholesterolemic Females via Reducing NO Production

**DOI:** 10.1155/2018/2543847

**Published:** 2018-04-11

**Authors:** Ying Luo, Quan-Neng Yan, Wan-Zhou Wu, Fan-Yan Luo

**Affiliations:** ^1^Department of Geriatric Medicine, Xiangya Hospital, Central South University, Changsha 410078, China; ^2^Department of Cardiology, Heart Center, Zhujiang Hospital, Southern Medical University, Guangzhou 510000, China; ^3^Department of Cardiothoracic Surgery, Xiangya Hospital, Central South University, Changsha 410078, China

## Abstract

Endothelial progenitor cells (EPCs) contribute to the endogenous endothelial repair program during hypercholesterolemia. EPC count and migratory and proliferative capacities remain unchanged in the premenopausal female with hypercholesterolemia. However, the changes of count and activity of circulating EPCs in the hypercholesterolemic postmenopausal females are unknown. Here, we find that the migratory and proliferative capacities of circulating EPCs were decreased in patients with hypercholesterolemia versus normocholesterolemia. No significant differences were found between postmenopausal females and age-matched males. NO production showed positive correlation with the activity and count of circulating EPCs in patients with hypercholesterolemia. Flow-mediated dilatation (FMD) is directly interrelated with EPC counts and function. Our findings reveal that decreased EPC count and endothelial dysfunction lead to less NO production in hypercholesterolemic postmenopausal females. Maintaining the EPC numbers and activity might be emerging as a potential therapeutic strategy to reduce the risk of cardiovascular injury in elder women.

## 1. Introduction

Endothelial progenitor cells (EPCs) are bone marrow-derived cells, participating in the process of endothelialization and vascular repair [1, 2]. Oxidized low-density lipoprotein (ox-LDL) decreases proliferation capacity and other cell functions such as migration, adhesion, and vasculogenesis [3–5]. Whereas, hypercholesterolemia, especially low-density lipoprotein (LDL), is a significant risk factor for cardiovascular disease resulting in endothelial dysfunction and occurrence of atherosclerosis [6, 7].

Cardiovascular diseases (CVD) as the predominant death cause in the world, with the increasing prevalence, manifest dissimilarly in males and females [8, 9]. Males develop CVD earlier versus females, but the overall lifetime risk of CVD is similar [10, 11]. At 55 years of age, the lifetime risk of first incident on coronary heart disease (CHD) in males is higher, but is lower on heart failure than females [10]. It suggests that estrogens might play a predominant role in CVD. Previous study has demonstrated that estrogen-treated EPCs possess higher capacity in migratory and tube forming in vitro, but the amount of EPCs is not affected [12]. Our preceding study has proved that the activity of circulating EPCs in premenopausal prehypertensive females is higher [13], but is impaired in postmenopausal prehypertensive females in our unpublished research. However, the count and activity of EPC, as well as whether flow-mediated dilatation (FMD) changes accordingly in postmenopausal hypercholesterolemic women, has been controversial and elusive.

EPC is involved in the occurrence and development of cardiovascular diseases. Various factors and conditions could affect its count and function. Patients with cardiovascular risk factors as age, sex, smoking, hypertension, diabetes mellitus, and dyslipidemia contain decreasing count and function of EPC [14]. In addition, EPC is regulated by cytokine such as NO, VEGF, GM-CSF, IL-8, and MCP-1 as well [15–20]. It has been reported that EPCs are impaired by TNF-*α* and IL-6 [21, 22]. Estrogen has been regarded as a stimulator for endothelial NO production and endothelial NO synthase (eNOS) activation and could lower the level of endogenous asymmetric dimethylarginin (ADMA) [23]. Fadini et al. have found that the number of EPCs is greater in fertile female than in male [24]. Our previous study has proved that there are more active EPCs in premenopausal prehypertensive females versus prehypertensive males of the same age. Moreover, the activity of EPCs correlated with the level of NO [13]. Based on these results, we determine the levels of NO, VEGF, GM-CSF, TNF-*α*, IL-6, IL-8, and MCP-1 to shed light the mechanism underlying the change of circulation in postmenopausal hypercholesterolemic females.

## 2. Methods

### 2.1. Study Population

Normocholesterolemic or hypercholesterolemic postmenopausal females and age-matched males were recruited (*n* = 20). Referring to classification of detection, evaluation, and treatment of high blood cholesterol in adults (ATP3), the patients with hyperlipidemia were diagnosed if serum total cholesterol (TC) > 5.18 mmol/L and low-density lipoprotein (LDL) > 3.36 mmol/L. The normocholesterolemic patients had no cardiovascular risk factors with TC < 5.18 mmol/L and LDL < 3.36 mmol/L. All patients were safe from cardiovascular disease as assessed by an intact medical history, physical examination, and blood tests before recruited in protocol. The patients with malignant disease, infection or inflammatory disorders, diabetes, and who are smokers were removed to avoid confounding factors affecting EPCs. Females with previous hysterectomy were excluded as well. The experimental protocol was ratified by the ethical committee of our hospital. The baseline characteristics of patients have been shown in Table 1.

Blood specimens were collected in the morning after overnight fasting, and the plasma was used for the determination of EPCs, TC, high-density lipoprotein (HDL), LDL, triglyceride (TG), plasma glucose, estradiol, and creatinine (Cr). Patients were banned from consuming alcohol or caffeine for 12 hours before the study. Drugs that might affect circulating EPCs, such as antiplatelet, anti-inflammatory, or antihypertension treatment, were not used.

### 2.2. Detection of Circulating EPC Count by Flow Cytometry Analysis and Cell Culture

Detection of EPCs has been noted in seminal studies [18, 25]. As mentioned, 100 *μ*L peripheral blood was immunostained with monoclonal antibodies against human CD34 (Becton Dickinson, Franklin Lakes, NJ, USA, PerCP-conjugated) and KDR (Sigma, St. Louis, MO, USA), followed by a PE-conjugated secondary antibody. Isotype-identical antibodies acted as controls (Becton Dickinson). After incubation, cells were lysed, washed with PBS, and fixed in 4% paraformaldehyde. And then, the analysis of 60,000 events took place after exclusion of debris and platelets. The number of circulating EPCs was judged by the ratio of CD34+KDR+ cells per 100 peripheral blood mononuclear cells (PBMNCs). To confirm the EPC phenotype, the attached cells were incubated with 1,1′-dioctadecyl-3,3,3′,3′-tetramethylindo-carbocyanine perchlorate-labeled acetylated LDL (DiI-acLDL, 10 *μ*g/mL, Molecular Probes) at 37°C for 1 h. Then, the cells were fixed with 4% paraformaldehyde for 30 min at 37°C and incubated with FITC-labeled Ulex europaeus agglutinin (lectin, 10 *μ*g/mL, Sigma) for 4 h at 37°C. After being stained, the samples were observed with a phase-contrast fluorescent microscope by two independent observers blinded to the study.

### 2.3. Migration and Proliferation Assay

As our earlier studies mentioned [26], a total of 2 × 10^4^ EPCs were isolated and resuspended in 250 *μ*L endothelial basal medium 2 (EBM2). It was pipetted in the upper chamber of a modified Boyden chamber (Costar Transwell assay, 8 *μ*m pore size, Corning, New York, USA). The chamber containing 500 *μ*L EBM-2 supplemented with 50 ng/mL VEGF was placed in a 24-well culture dish. Transmigrated cells were counted by two independent investigators blinded after 24 h incubation at 37°C. EPC proliferation was assessed using 3-(4,5-dimethylthiazol-2-yl)-2,5-diphenyltetrazolium bromide (MTT) assay. EPCs were digested with 0.25% trypsin before being cultured for 7 days. And then it was cultured in a serum-free medium in a 96-well culture plate (200 *μ*L/well). EPCs were cultured for 24 h before being supplemented with 10 *μ*L MTT (5 g/L, Fluka, Sigma-Aldrich, St. Louis, Missouri, USA) and incubated for another 4 h. The supernatant was aspirated and discarded. Then, after shaking the EPC preparation with 200 *μ*L dimethyl sulfoxide for 10 min, the optical density (OD) value was measured at 490 nm.

### 2.4. Measurement of Levels of NO, VEGF, GM-CSF, TNF-*α*, and IL-6 in Plasma

Greiss method was used to ascertain nitrite, the stable metabolite of NO. The formation of nitrite (NO2-) and nitrate (NO3-) was measured in cell culture supernatants. The results are presented as *μ*mol NOx of NO3-/NO2- per liter of medium. According to the manufacturer's instructions, high-sensitivity enzyme-linked immunosorbent assay (R&D Systems, Wiesbaden, Germany) was used to measure the levels of VEGF, GM-CSF, TNF-*α*, and IL-6 in the plasma.

### 2.5. Measurement of NO, VEGF, GM-CSF, TNF-*α*, IL-6, IL-8, and MCP-1 in the Conditioned Medium from EPCs

The identified cultured EPCs were switched to the DMEM/20% fetal bovine serum (no supplemental growth factors) for 48 hours, and then ELISA was used to assay the conditioned media for NO, VEGF, GM-CSF, TNF-*α*, IL-6, IL-8, and MCP-1 as mentioned previously [18].

### 2.6. Flow-Mediated Dilatation

The measurement of FMD was performed as previously reported [27, 28]. Brachial artery FMD was evaluated by single trained investigator with high-resolution ultrasonography using a 5–12 MHz linear transducer on an HDI 5000 system (Washington, USA). After 15 min rest, the brachial artery was studied 20 to 100 mm proximal to the antecubital fossa in supine patients. Pressure in an upper-forearm sphygmomanometer cuff was raised to 250 mmHg for 5 min. FMD was calculated automatically as the percentage increase in mean diastolic diameter reactive hyperaemia 55 to 65 s after deflation to baseline. After a further 15 min, 400 *μ*g sublingual glyceryl trinitrate (GTN) was given and diastolic diameter was remeasured after 5 min for measurement of endothelial-independent dilatation.

### 2.7. Statistical Analysis

SPSS version 11.0 (SPSS Inc., Chicago, IL, USA) was used to analyze the data which were presented as mean value ± standard deviation. Two-factor analysis of variance (sex and status of ortholiposis or hypercholesterolemia) was used to analyze comparisons among four groups. When indicated by a significant *F* value, the Newman-Keuls method as a post hoc test was used to identify differences among mean values. Pearson's coefficient (*r*) was used to calculate univariate correlations. Statistical significance was assumed if *p* value < 0.05.

## 3. Results

### 3.1. Clinical Baseline Characteristics

The general characteristics of the study population were shown in Table 1. The four groups were similar in terms of age, BMI, blood pressure, heart rate, and fasting plasma glucose (FPG). LDL and TC were significantly higher in hypercholesterolemic groups versus normocholesterolemic groups. No significant difference was found between the females and the males with hypercholesterolemia or ortholiposis. Also, the levels of TG, HDL, and estradiol did not significantly differ between the females and males. FMD was lower in hypercholesterolemic males related with normocholesterolemic, but no difference between hypercholesterolemic and normocholesterolemic females.

### 3.2. Count and Activity of Circulating EPCs

**Figure 1:**
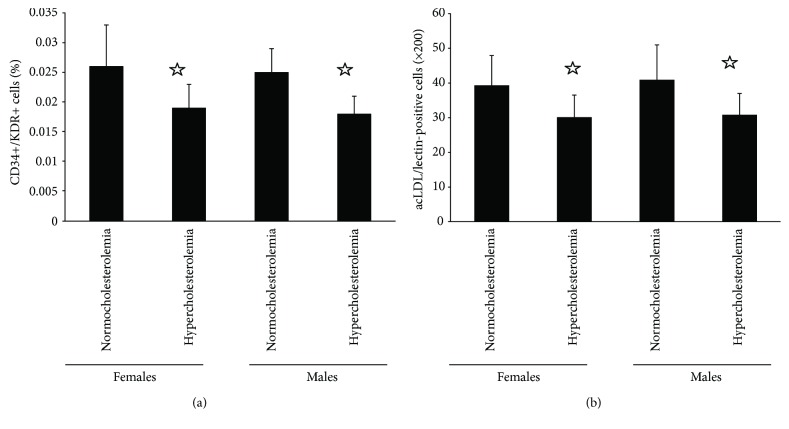
The amount of circulating EPCs. Evaluated by (a) FACS analysis and (b) phase-contrast fluorescent microscope, the amount of circulating EPCs in normocholesterolemic and hypercholesterolemic males was almost equal to those in normocholesterolemic and hypercholesterolemic postmenopausal females. The EPC number in hypercholesterolemic males or postmenopausal women was lower than that in normocholesterolemic males or postmenopausal females. Data are given as mean ± SD. ^☆^*p* < 0.05 versus normocholesterolemia in the same sex group.

The amount of circulating EPCs in hypercholesterolemic females and males was less than in normocholesterolemic females and males, respectively. No difference was found between women and men with similar situation of blood cholesterol (Figure 1(a)). Cell culture assay exhibited more EPCs in normocholesterolemic females or males than in hypercholesterolemic females or males, respectively. Also, no sex difference was found (Figure 1(b)).

**Figure 2:**
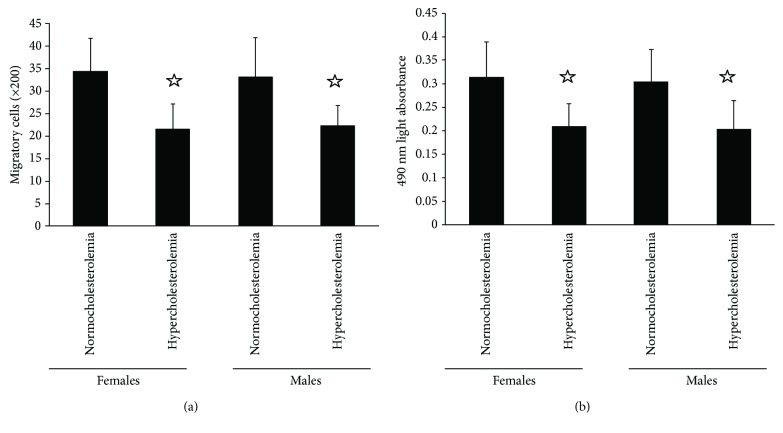
The activity of circulating EPCs. The migratory (a) and proliferative (b) activities of circulating EPCs in normocholesterolemic and hypercholesterolemic males were equal to those in normocholesterolemic and hypercholesterolemic postmenopausal females. The EPC function in hypercholesterolemic males or postmenopausal females was lower than that in normocholesterolemic males or postmenopausal females. Data are given as mean ± SD. ^☆^*p* < 0.05 versus normocholesterolemia in the same sex group.

The migratory activity of EPCs in hypercholesterolemic females and males decreased than that in normocholesterolemic ones (Figure 2(a)). Analogously, the proliferative activity of circulating EPCs in normocholesterolemic females and males was higher than that in hypercholesterolemic ones (Figure 2(b)). Neither normocholesterolemia nor hypercholesterolemia showed sexual difference in migratory activity or proliferative activity of EPCs.

### 3.3. Plasma Levels of NO, VEGF, GM-CSF, TNF-*α*, and IL-6

**Figure 3:**
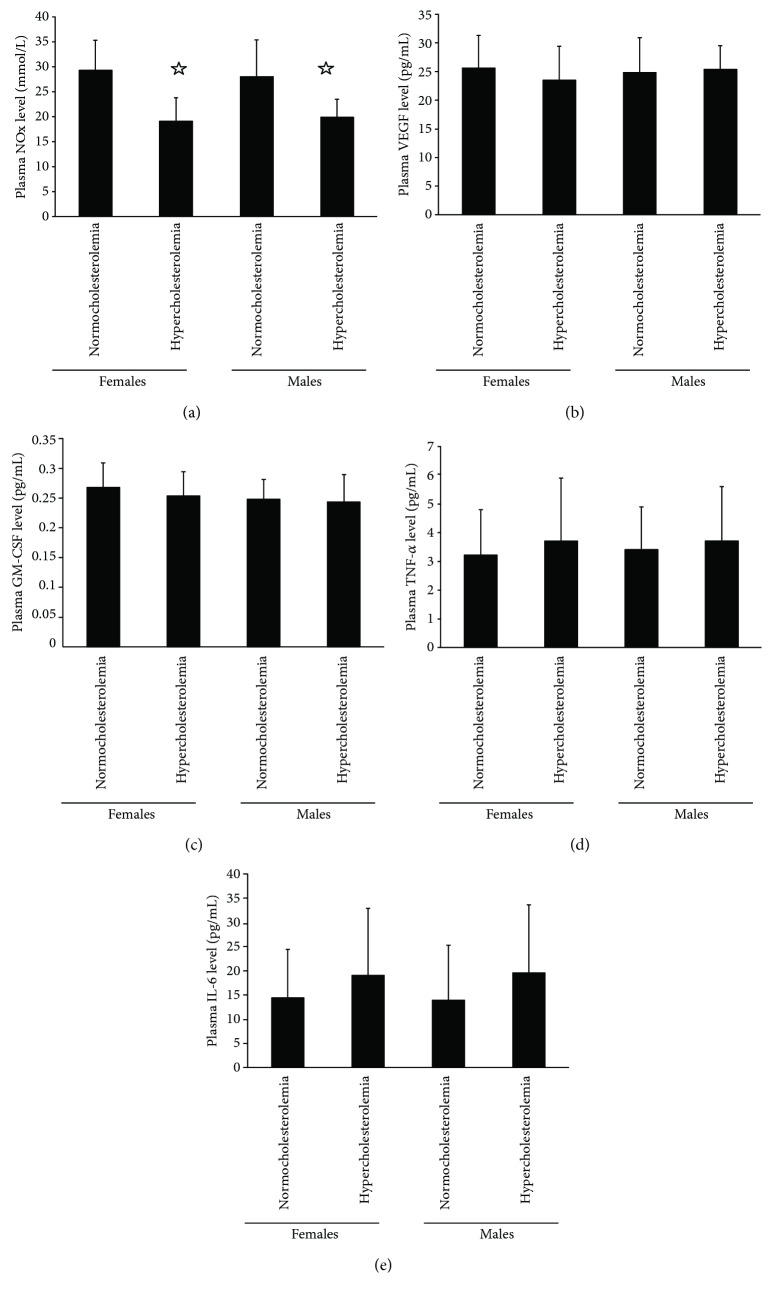
The plasma NO, VEGF, GM-CSF, TNF-*α*, and IL-6 levels. (a) The plasma NO level in normocholesterolemic and hypercholesterolemic males was equal to that in normocholesterolemic and hypercholesterolemic postmenopausal females. The plasma NO level in hypercholesterolemic males or postmenopausal females was lower than that in normocholesterolemic males or postmenopausal females. (b, c) There was no significant difference in the plasma VEGF and GM-CSF level between the four groups. (d, e) There was no significant difference in the plasma TNF-*α* and IL-6 level between the four groups. Data are given as mean ± SD. ^☆^*p* < 0.05 versus normocholesterolemia in same sex group.

The levels of NO in normocholesterolemic females and males were higher than that in hypercholesterolemic ones (Figure 3(a)). However, no difference was found about the levels of VEGF, GM-CSF, TNF-*α*, and IL-6 in plasma among the four groups (Figures 3(b)–3(e)). Similarly, no sexual differences were found among the four groups.

### 3.4. NO, VEGF, GM-CSF, TNF-*α*, IL-6, IL-8, and MCP-1 Secretion by EPCs

**Figure 4:**
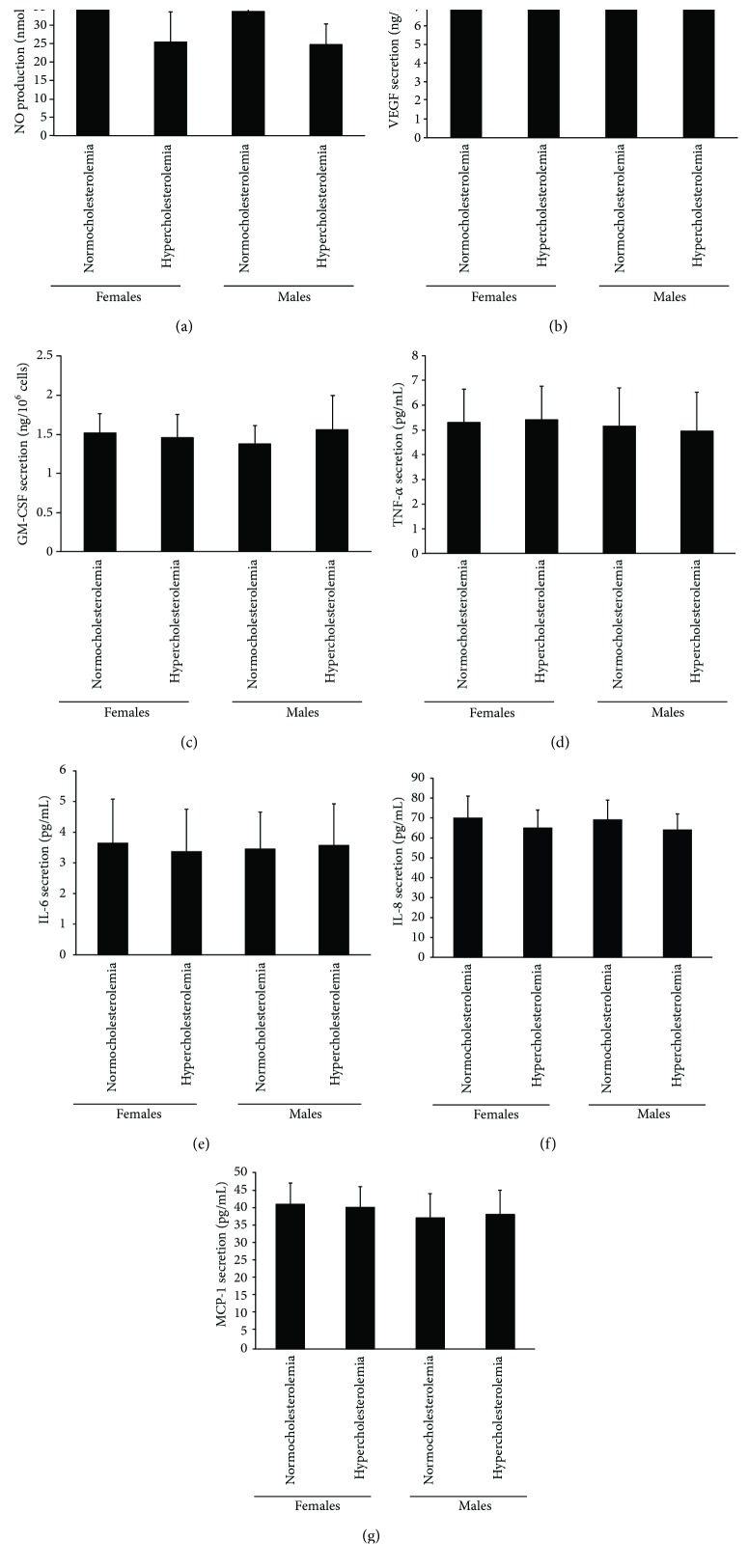
The NO, VEGF, GM-CSF, TNF-*α*, IL-6, IL-8, and MCP-1 secretion by EPCs. (a) The NO secretion by EPCs in normocholesterolemic and hypercholesterolemic males was equal to that in normocholesterolemic and hypercholesterolemic postmenopausal females. The plasma NO level in hypercholesterolemic males or postmenopausal females was lower than that in normocholesterolemic males or postmenopausal females. (b, c) There was no significant difference in VEGF and GM-CSF secretion by EPCs between the four groups. (d–g) There was no significant difference in TNF-*α*, IL-6, IL-8, and MCP-1 secretion by EPCs between the four groups. Data are given as mean ± SD. ^☆^*p* < 0.05 versus normocholesterolemia in same sex group.

Analogous to the distribution of plasma NO level, the NO secretion by cultured EPCs in normocholesterolemic females and males was higher than that in hypercholesterolemic ones (Figure 4(a)). Similarly, the VEGF, GM-CSF, TNF-*α*, and IL-6 in the CM from EPCs showed no significant difference in the four groups (Figures 4(b)–4(e)). No difference in the four groups was found in the secretion of IL-8 or MCP-1 by EPCs (Figures 4(f) and 4(g)).

### 3.5. Correlation Analysis between FMD and EPCs or NO

**Figure 5:**
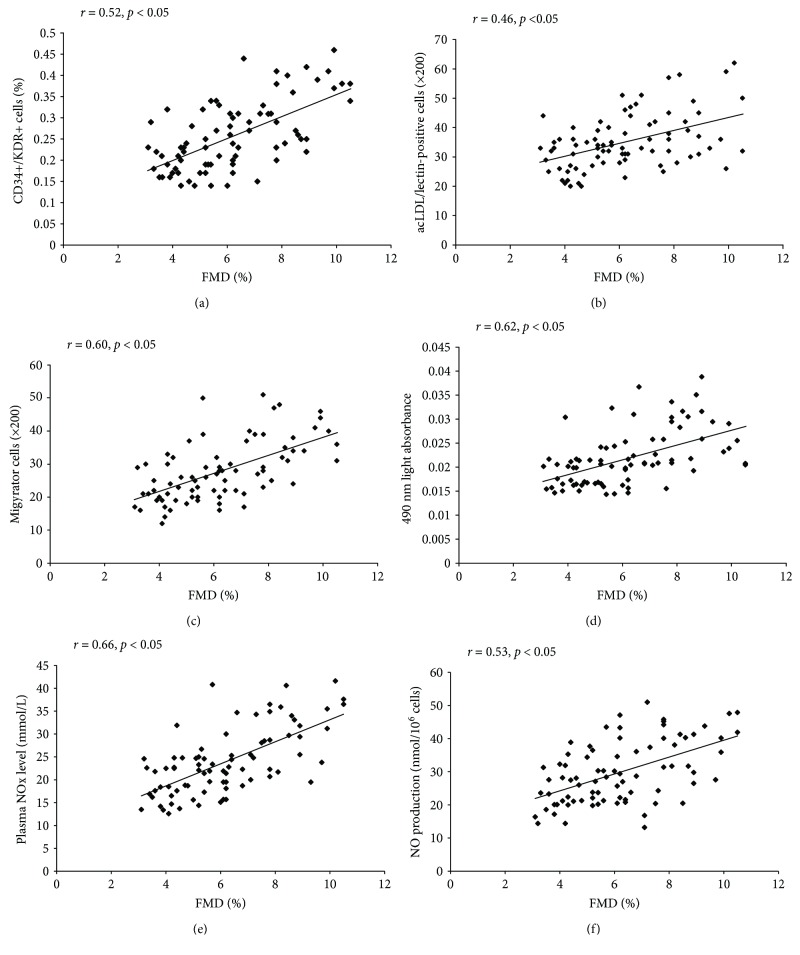
The correlation between circulating EPCs or NO level and FMD. The amount of circulating EPCs evaluated by FACS (a) or by cell culture (b) correlated with the FMD. There was a correlation between the EPC proliferation (c) or migration (d) and FMD. In addition, there was a correlation between the plasma NO level (e) or NO secretion by EPCs (f) and FMD.

As shown, a strong univariate correlation was found between FMD and the amount of EPCs evaluated by flow cytometry analysis and by cell culture (Figures 5(a) and 5(b)). Also, the migratory and proliferative activities of EPCs positively correlated with FMD (Figures 5(c) and 5(d)). In addition, the level of NO in plasma and NO secretion by EPCs exhibited positive impact on FMD (Figures 5(e) and 5(f)).

## 4. Discussion

In the present study, we identified the decreased number and activity of circulating EPCs in patients with hypercholesterolemia when compared with normocholesterolemic subjects. The NO level in plasma or conditioned medium in hypercholesterolemic patients was lower as well. Similarly, a significant correlation between EPCs and plasma NO level as well as NO secretion by EPCs was ascertained. No sex difference was found in the number and activity of circulating EPCs in hypercholesterolemic or normocholesterolemic postmenopausal females compared with age-matched males. Correlation analysis manifested that endothelial function was correlated with the count and activity of EPCs, as well as NO production. No evidence in the present study demonstrated that VEGF, GM-CSF, IL-6, IL-8, MCP-1, and TNF-*α* were involved in hypercholesterolemia-induced EPC dysfunction.

The results showed decreased amount and activity of circulating EPCs, as well as FMD, in postmenopausal hypercholesterolemic patients versus subjects with normocholesterolemia. It indicated that hypercholesterolemia could decrease EPC number and impair EPC function and further led to endothelial dysfunction. We found that, compared with hypercholesterolemic men of the same age, the amount and activity of EPCs were preserved in premenopausal females with hypercholesterolemia (data not shown). However, no sex difference was found when postmenopausal females were compared with males of the same age. It has been reported that the levels of circulating EPCs are inversely associated with the occurrence and development of cardiovascular events which were relatively rare in middle-aged premenopausal females [29, 30]. In murine models, 17*β*-estradiol delivery promoted the mobilization and proliferation of EPCs, enhanced reendothelialization, and attenuated neointima formation after vascular injury [31, 32]. Therefore, hypercholesterolemia-related circulating EPC decline and endothelial dysfunction in postmenopausal females might be associated with decreasing endogenous endothelial repair capacity because of lacking estrogen protection.

The requirement for NO in estrogen-induced regulation of EPCs has already been reported in murine models [33]. The NO secreted by EPCs is related to endothelial repair capacity of EPCs [34, 35]. Similarly, our study showed that compared to normocholesterolemic females and males, the level of NO in plasma and NO secretion by EPCs was lower in postmenopausal hypercholesterolemic females and males of the same condition. Thus, indicating the reduced NO by EPCs might mediate the decrease of the amount and function of EPCs in postmenopausal hypercholesterolemic females. There was no significance difference in the level of VEGF, GM-CSF, TNF-*α*, IL-6, IL-8, and MCP-1 between normocholesterolemic and hypercholesterolemic males and females, supporting that the weakened endothelial repair capacity in postmenopausal hypercholesterolemic females is independent of alterations in the above cytokines.

NO is a biologically active radical, synthesized in vascular endothelial cells through eNOS, depending on the balance between NO production and inactivation [36]. The NO pathway plays a critical role in modulating EPC function, as well as in vascular repair in response to injury [37]. Similarly, corelationship analysis in our present and previous study showed that the amount and activity of EPCs were associated with endothelial function [13]. The decreasing level of NO in plasma and NO production by EPC was causative for impaired endothelial function. In contrast, increasing NO production improved endothelial function. All of the results indicated EPC dysfunction and decreased NO level correlated with vascular endothelial dysfunction.

Clinically, the amount and activity of EPCs might reflect the endothelial integrity and repair capacity which have been regarded as a novel biomarker of endothelial function and cardiovascular diseases. Patients with persistent hyperglycemic metabolism exhibit a high risk of developing CVD. In vitro, the cultivation of EPCs in hyperglycemic medium imitating the in vivo situation leads to a decline in eNOS phosphorylation associated with a decrease in NO production [38]. Hypertension has been proven to be the strongest independent predictor of EPV functional decline in patients with CAD [39]. A series of ensuing studies have supported the significance of the relation between EPC and hypertension [40, 41]. The count and migratory capacity of circulating EPCs of patients with coronary artery disease are lower than age-matched healthy subjects or individual with high serum LDL cholesterol levels [39]. In addition, the EPC count is linked to endothelium-dependent vasodilatation, coronary collateral circulation, risk of a first major cardiovascular event, and revascularization surgery [29, 40, 42]. Whereas, compared to premenopausal hypercholesterolemic females, the decreased EPC count and activity in postmenopausal females with hypercholesterolemia indicate the endothelial dysfunction. Meanwhile, the NO production by EPC is significantly reduced and leads to endothelium injury. Consequently, taking into consideration the upregulation of EPC count and activity may benefit for the elder females with hypercholesterolemia. It may rescue and ameliorate endothelial function and reduce risk of cardiovascular event. Evidences showed that ox-LDL could reduce EPC count and impair EPC migration and proliferation through exerting deleterious influences on the PI3k/PKB/Akt/eNOS/NO signaling cascade [38, 43–45]. However, exercise, drugs such as statins, erythropoietin, estrogens, and VEGF could increase circulating EPC count and proliferation and migration as the activators of the PI3K/Akt pathway [46, 47]. In conclusion, the present study demonstrated that there is no sexual difference in circulating EPC count and endothelial function in hypercholesterolemic postmenopausal females and age-matched males, which might be linked to NO production. The weakened endogenous endothelial repair capacity may offer part of the explanation for decreased endothelial protection after lacking enough estrogen. This finding offers a novel target to recover endothelial function in postmenopausal females with hypercholesterolemia.

## Figures and Tables

**Table 1:** Clinical and biochemical characteristics.

Characteristics	Normocholesterolemic females (*N* = 20)	Hypercholesterolemic females (*N* = 20)	Normocholesterolemic males (*N* = 20)	Hypercholesterolemic males (*N* = 20)
Age (years)	55.1 ± 2.5	56.2 ± 3.0	56.9 ± 3.3	54.9 ± 2.7
Height (cm)	162.4 ± 5.0	162.1 ± 6.1	168.5 ± 6.5^#^	167.8 ± 6.5^#^
Weight (kg)	61.9 ± 4.0	63.3 ± 5.4	66.6 ± 5.9^#^	67.4 ± 4.7^#^
BMI (kg/cm^2^)	23.5 ± 1.4	24.1 ± 2.0	23.4 ± 1.2	23.9 ± 1.4
SBP (mmHg)	126.9 ± 9.7	123.5 ± 9.7	128.3 ± 5.7	124.6 ± 7.7
DBP (mmHg)	79.4 ± 8.0	76.7 ± 6.7	80.9 ± 4.9	78.3 ± 6.4
HR (beats/min)	76.9 ± 9.2	75.4 ± 8.2	74.3 ± 9.0	77.4 ± 9.8
AST (mmol/L)	22.0 ± 5.5	22.7 ± 4.9	23.5 ± 4.3	22.1 ± 5.4
ALT (mmol/L)	19.7 ± 5.5	21.3 ± 4.8	21.4 ± 7.5	20.6 ± 5.8
BUN (mmol/L)	4.6 ± 0.9	4.4 ± 0.6	4.7 ± 0.7	4.5 ± 0.7
Cr (mmol/L)	53.3 ± 8.5	51.3 ± 11.9	56.8 ± 9.7	50.6 ± 9.0
LDL (mmol/L)	2.68 ± 0.33	4.20 ± 0.44^☆^	2.71 ± 0.32	4.30 ± 0.52^☆^
TC (mmol/L)	4.50 ± 0.48	6.06 ± 0.46^☆^	4.51 ± 0.40	6.21 ± 0.40^☆^
HDL (mmol/L)	1.31 ± 0.17	1.25 ± 0.12	1.28 ± 0.17	1.26 ± 0.16
TG (mmol/L)	1.49 ± 0.14	1.55 ± 0.10	1.51 ± 0.13	1.53 ± 0.12
FPG (mmol/L)	4.80 ± 0.47	4.62 ± 0.56	4.82 ± 0.47	4.71 ± 0.44
FMD (%)	7.65 ± 1.51	5.02 ± 1.10^☆^	7.44 ± 1.78	4.69 ± 1.35^☆^

BMI: body mass index; SBP: systolic blood pressure; DBP: diastolic blood pressure; HR: heart rate; AST: aspartate transaminase; ALT: alanine aminotransferase; BUN: blood urea nitrogen; Cr: creatinine; LDL: low-density lipoprotein; TC: total cholesterol; HDL: high-density lipoprotein; TG: triglyceride; FPG: fasting plasma glucose; FMD: flow-mediated dilatation. Data are given as mean ± SD. ^☆^*p* < 0.05 versus normotension in the same sex group; ^#^*p* < 0.05 versus postmenopausal females.
